# Disrupted Amygdala Connectivity Is Associated With Elevated Anxiety in Sensorineural Hearing Loss

**DOI:** 10.3389/fnins.2020.616348

**Published:** 2020-12-10

**Authors:** Tian-Yu Tang, Ying Luan, Yun Jiao, Jian Zhang, Sheng-Hong Ju, Gao-Jun Teng

**Affiliations:** Jiangsu Key Laboratory of Molecular and Functional Imaging, Department of Radiology, Zhongda Hospital, Medical School of Southeast University, Nanjing, China

**Keywords:** sensorineural hearing loss, amygdala, orbitofrontal cortex, striatum, emotion, functional connectivity, uncinate fasciculus

## Abstract

**Background and Purpose:** Hearing loss is associated with rising risks of emotional impairments, suggesting emotional processing networks might be involved in the neural plasticity after hearing loss. This study was conducted to explore how functional connectivity of the amygdala reconfigures in the auditory deprived brain and better understand the neural mechanisms underlying hearing loss-related emotional disturbances.

**Methods:** In total, 38 chronic sensorineural hearing loss (SNHL) patients and 37 healthy controls were recruited for multimodal magnetic resonance imaging scanning and neuropsychological assessments. Voxel-wise functional connectivity (FC) maps of both the left and right amygdala were conducted and compared between the SNHL patients and healthy controls. The uncinate fasciculus (UF), an association fiber pathway, was reconstructed in both groups. The track number, mean track length, fractional anisotropy (FA) and mean diffusion values of the left and right UF were further quantified, respectively. Besides, Pearson's correlation analyses were conducted to investigate the relationship between the functional/structural abnormalities and the negative emotional states in SNHL patients.

**Results:** The SNHL patients presented higher depressive and anxious levels compared to the healthy controls. Decreased FCs were detected between the amygdala and the auditory cortex, striatum, multimodal processing areas, and frontoparietal control areas in the SNHL patients. The amygdala was found to be structurally connected with several FC decreased regions through the UF. Moreover, the hypo-synchronization and the white matter impairment were both found to be associated with patients' elevated anxious status.

**Conclusions:** These functional and structural findings depicted the reconfiguration of the amygdala in SNHL, which provided a new perspective toward the functional circuit mechanisms targeting the emotional impairments related to hearing loss.

## Introduction

Hearing loss was reported to be associated with increased risks of developing emotional disorders, such as depression and anxiety (Contrera et al., 2017), with profound impacts on mental health and the quality of life. Higher odds of anxiety were found in both mild and moderate hearing impairment patients (Contrera et al., 2017), with odds ratios of 1.32 and 1.59, respectively. In older adults, hearing loss was associated with greater odds of depression (Lawrence et al., 2019). The association between hearing loss and emotional dysfunctions indicated that emotional processing circuits might be involved in the wide-ranging reorganization after hearing loss. Therefore, hearing loss might play a critical role in neural plasticity and contribute to dramatic neuro-plastic alterations in the brain.

The amygdala, which consists of a group of nuclei with complex connections with multiple brain areas across the whole brain, is a crucial structure in reward and emotional responses (Janak and Tye, 2015), and is implicated in many affective disorders. The amygdala receives direct neural projections from thalamic and cortical levels of the central auditory pathway and exerts direct projections to the auditory midbrain (Amaral et al., 1992; Sah et al., 2003). Sound can influence the function of the amygdala, and in turn, the amygdala can also modulate the neural activity or plasticity in the auditory system (Kraus and Canlon, 2012). Emerging evidence has indicated that hearing loss causes abnormal neural responses in the amygdala (Husain et al., 2014; Sheppard et al., 2014; Chen et al., 2016). However, the intrinsic long-ranging connectivity pattern of the amygdala and its relationships with the amygdala-related emotional processing remains far from clear.

The functional interactions of the spatially distributed human brain regions could be revealed with imaging techniques. Neuroimaging studies have widely documented amygdala dysconnectivity in many emotional disorders (Marchand, 2010; Moses-Kolko et al., 2010). A recent study on tinnitus patients suggested that amygdala-cortical functional connectivity (FC) with the prefrontal-cingulate-temporal circuit could provide evidence to underlying neuropathological mechanisms of tinnitus-induced depressive disorder (Chen et al., 2017). However, the alternated FC of the amygdala with corresponding brain regions remains unclear in sensorineural hearing loss (SNHL) patients.

The uncinate fasciculus (UF), an association fiber pathway, was reported to be directly or indirectly involved in auditory processing (Chern et al., 2020). The amygdala and the pre-frontal cortex, known as two key regions involved in emotion processing, were structurally connected through the UF (Hau et al., 2016; Hein et al., 2018). Although lower white matter microstructural integrity of the UF was found in hearing loss patients (Croll et al., 2020), the structural alterations in neural plasticity of the hearing loss remain unclear. The above findings provided evidence to investigate the relationship between white matter integrity of the UF with the amygdala related emotional impairments in hearing loss.

In this study, we combined the resting-state functional magnetic resonance imaging (rs-fMRI) and diffusion tensor imaging (DTI) techniques to investigate the potential connectivity alterations between the amygdala with the whole brain and interoperate how this disruption is associated with abnormal emotional states in SNHL patients.

## Materials and Methods

### Participants

Thirty-eight long-term SNHL patients and 37 age-, gender-, and education-matched healthy controls were recruited in this study. All the participants are right-handed. All the SNHL patients had a consistent bilateral post-lingual hearing loss for at least the past 3 years, with the mean hearing thresholds above 25 dB for both ears. Only one of the patients declared an etiology of ototoxic drug application; others have no exact causes of their hearing loss.

Any participant has self-reported tinnitus; acoustic neuroma or Meniere's disease; poorly controlled diabetes or hypertension; a clinical history of cancer, head injury, stroke or otologic surgery; Alzheimer's disease, schizophrenia, seizures, and other neuropsychiatric diseases; and inability to undergo the MRI scanning would be excluded from this study.

Hamilton Depression Rating Scale (HAMD) and Self-Rating Anxiety Scale (SAS) were used to evaluate the depressive and anxious levels. All the participants provided written informed consent before the experiment. The current study was approved by the Ethics Committee of Affiliated Zhongda Hospital of Southeast University, and all the procedures were performed following the Declaration of Helsinki.

### Audiological Assessment

The hearing thresholds at the frequency of 0.25, 0.5, 1, 2, 4, and 8 kHz for each participant were measured via pure tone audiometry. The pure-tone average (PTA) of each ear was calculated by averaging the air conduction thresholds at 0.5, 1, 2, and 4 kHz. The binaural PTA was determined as the mean value of the monaural PTA. The participant with conductive deafness would be identified and excluded according to the acoustic immittance test. In the control group, all the participants had the PTA ≤ 25 dB hearing level (HL) for each ear. In the SNHL group, all participants had the PTA > 25 dB HL for each ear.

### MRI Acquisition

Image scanning was performed using a Siemens 3.0 T MRI scanner (Siemens, Erlangen, Germany) with a 12-channel head coil. A headphone, as well as earbuds, were used to alleviate the noise during scanning. During the MRI scanning procedure, the participants were asked to keep the head still, eyes closed, and not think anything.

The high-resolution three-dimensional magnetization-prepared rapid gradient-echo (3D MPRAGE) T1-weighted sequence were used to acquired structural image: repetition time (TR) = 1,900 ms, echo time (TE) = 2.48 ms, inversion time = 900 ms, flip angle = 9.0°, slice number = 176, slice thickness = 1.0 mm, field of view (FOV) = 250 × 250 mm, matrix = 256 × 256.

Resting-state functional MRI (rs-fMRI) images were acquired with a gradient-recalled-echo echo-planar imaging (GRE-EPI) as follows: TR = 2,000 ms, TE = 13 ms, flip angle = 90.0°, slice number = 32, slice thickness = 4.0 mm, FOV = 240 × 240 mm, matrix = 64 × 64, volumes = 240.

The DTI raw data were acquired using a single-shot echo planar imaging sequence (EPI) with the following parameters, repetition time: 10,000 ms, echo time: 95 ms, flip angle: 90, slice number: 31, slice thickness: 2.0 mm, spacing between slices: 2.0 mm, b-values: 0 and 1,000 s/mm^2^, 30 volumes with non-collinear directions, matrix: 128 × 128.

### Data Pre-processing

All the functional data were pre-processed using the Statistical Parametric Mapping software (SPM12, https://www.fil.ion.ucl.ac.uk/spm/) and the Data Processing and Analysis for Brain Imaging toolbox (DPABI V4.3, http://rfmri.org/dpabi/). The first 10 volumes of the EPI images were discarded to obtain steady states. The remaining images underwent the slice-timing adjustment and realignment for the head motion correction. Six head motion parameters (three translations and three rotations) were used for head motion correction (Jenkinson et al., 2002).

Subjects whose head motion exceeded 2.0 mm of translation in the x, y, z plane, or 2.0 degrees of axial rotation would be excluded from this study (Cui et al., 2014). In this study, all the participants were with the head motion below the thresholds. Of note, the SNHL group and control group did not show significant differences in the head motion determined by the frame-wise displacement (FD, *t* = 0.4678, *P* = 0.6413), a summary statistic of the head motion (Power et al., 2012). The individual functional images were normalized to a standard template in MNI space via a DARTAL method and resampled into the voxel size of 3 × 3 × 3 mm^3^. It has been well-known that the head motion has profound influences on the rs-fMRI signal. Head motion parameters using a Friston 24-parameter model were also regressed. Then linear detrending was performed. The cerebrospinal fluid and white matter signals were regressed from the time series. The time series following confound regressions underwent the band-pass filtering (0.01 to 0.08 Hz) (Zou et al., 2009). The normalized images were further spatially smoothed with a 6-mm full-width half-maximum (FWHM) Gaussian kernel.

The diffusion data pre-processing was performed based on the DSI studio (http://dsi-studio.labsolver.org). The following steps were performed, DICOM raw data were reconstructed, motion and eddy current correction was performed, quality control routines were conducted (Yeh et al., 2019b), b-table was checked by an automatic quality control routine to ensure its accuracy (Schilling et al., 2019). The diffusion data were reconstructed in the MNI space using q-space diffeomorphic reconstruction to obtain the spin distribution function (Yeh et al., 2010). A diffusion sampling length ratio of 1.25 was used, and the output resolution was set to be 2 mm isotropic. The restricted diffusion was quantified using restricted diffusion imaging (Yeh et al., 2017).

### Voxel-Wise Functional Connectivity Analyses

The voxel-wise FC analysis was employed using the Resting-State fMRI Data Analysis Toolkit (REST V1.8) toolbox. The left and right amygdala were set as seed regions based on the Anatomical Automatic Labeling (AAL) template (Tzourio-Mazoyer et al., 2002). Pearson's correlation coefficients between the mean time courses of the left and right amygdala with the whole brain were calculated. Fisher's r-to-z transformation was applied to generate FC *z*-score maps. Two sample *t*-test was applied to determine the between-group differences of voxel-wise FC maps, with age, gender, and education as the covariates. The resulting statistic maps were further thresholded at the false discovery rate (FDR) corrected *P* < 0.05 and with the cluster size over 30 voxels.

### White Matter Reconstruction

A deterministic fiber tracking algorithm was conducted based on the DSI studio with improved tracking strategies (Yeh, 2020). The tractography atlas was used to map the bilateral UF with a distance tolerance of 16 mm (Yeh et al., 2018). A seeding region was placed at the track region indicates by tractography atlas. A ROA was placed at the track tolerance region with a cubic volume size of 1.5e + 06 mm^3^. The track-to-voxel ratio was set to 2. The anisotropy threshold was randomly selected. The angular threshold was randomly selected from 15 to 90 degrees. The step size was randomly selected from 0.5 to 1.5 voxels. Tracks with a length shorter than 30 or longer than 300 mm were discarded from the fiber reconstruction results. To remove false connections, topology-informed pruning was applied with 32 iterations (Yeh et al., 2019a). Finally, after the bilateral UF were identified, the number of tracts, mean length of the tracks, mean FA and MD values along the left and right UF were calculated for further analyses, respectively.

### Statistical Analyses

All analyses were performed using the freely available R statistical software package (version 3.0.1; http://www.Rproject.org). Data are presented as the means [standard deviations (SDs)] and numbers (percentages), as appropriate. Between-group differences in age, gender, education, PTA, and neuropsychological assessments were evaluated with two sample *t*-test, Mann-Whitney *U*-test, or chi-square test as appropriate, as listed in Table 1. For FCs and fiber track measurements, general linear models were used to test for main effects between the SNHL and healthy controls, controlled by age, gender, and years of education for each participant. To determine the relationship between abnormal amygdala connectivity and emotional status in the patients with SNHL, partial correlations between radiological measures and clinical variables were calculated, also controlled by age, gender, and years of education.

**Table 1:** Demographical, clinical, and neuropsychological characteristics of the patients with SNHL and healthy controls.

	**SNHL patients**	**Healthy controls**	***P-*value**
	**(*n* = 38)**	**(*n* = 37)**	
Age (years)^a^	54.11 ± 9.25	52.51 ± 9.25	0.552
Gender (male)^b^	23(60.5%)	18(48.7%)	0.302
Education (years)^c^	11.37 ± 3.29	12.03 ± 3.36	0.394
Handedness (right)	38(100%)	37(100%)	
PTA of right ear (dB HL)^c^	43.27 ± 19.29	19.15 ± 4.14	<0.001^**^
PTA of left ear (dB HL)^c^	48.23 ± 22.59	19.19 ± 5.27	<0.001^**^
Mean PTA of both ears (dB HL)^c^	45.75 ± 18.37	19.17 ± 4.40	<0.001^**^
HAMD^c^	6.08 ± 3.88	3.81± 2.17	0.011^*^
SAS^c^	31.45 ± 7.76	28.19 ± 5.84	0.043^*^

*All the data were presented as mean ± standard deviation for continuous variables, and as percentages for categorical variables*.

* *P < 0.05*,

** *P < 0.001*;

*PTA, pure tone audiometry from 0.5, 1, 2, and 4 kHz; dB HL, decibel hearing level; HAMD, Hamilton Depression Rating Scale; SAS, Self-Rating Anxiety Scale*.

a *two sample t-test*;

b *chi-square test*;

c *Mann-Whitney U-test*.

## Results

### Demographic, Hearing, and Neuropsychological Characteristics

**Figure 1:**
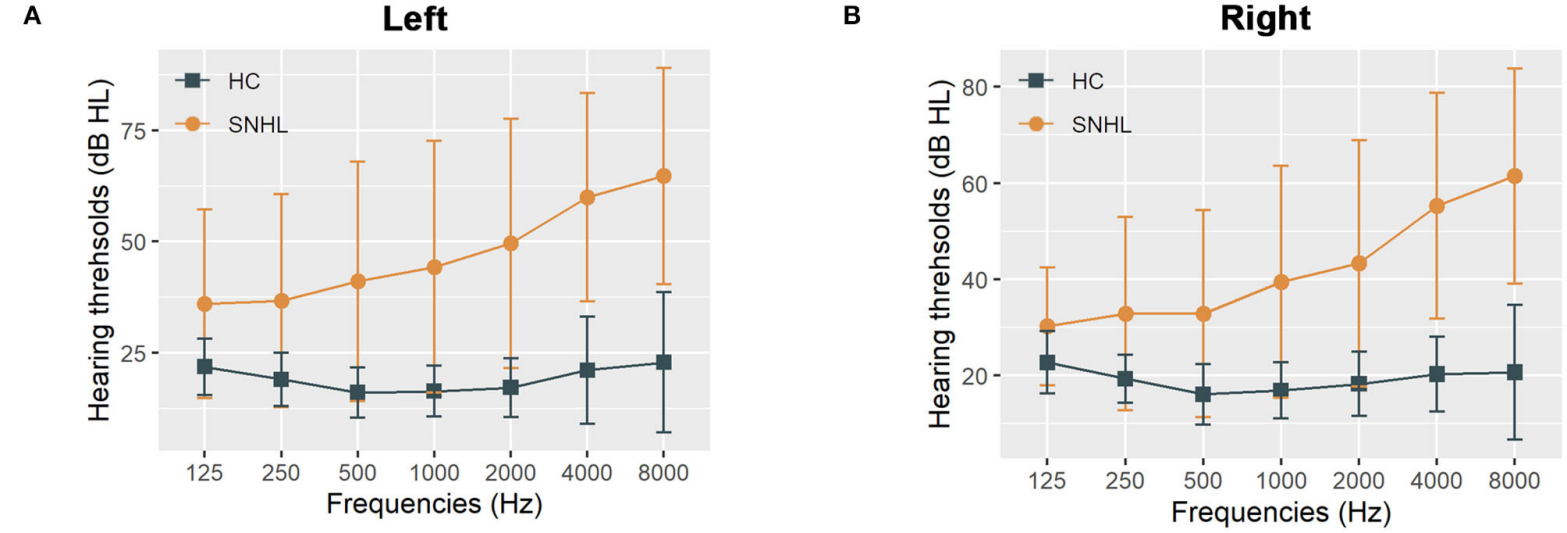
The hearing thresholds of the SNHL and control groups. **(A)** The hearing thresholds the left ear in the SNHL and control groups; **(B)** The hearing thresholds the right ear in the SNHL and control groups. The data are shown as mean ± standard deviation. HL, hearing level; SNHL, sensorineural hearing loss; HC, healthy controls.

The detailed clinical characteristics were summarized in Table 1. The SNHL patients were well-matched in age, gender, education, and handedness with the healthy controls. The PTA for both sides in the control group were within the normal range ( ≤ 25 dB HL). The mean duration of auditory deprivation in the SNHL subjects was 10.21± 8.79 years. Compared with the healthy controls, SNHL patients were associated with higher monaural PTAs and binaural mean PTAs, which exceeded the normal range, shown in Figure 1. The patients with SNHL showed significantly increased HAMD (*P* = 0.043) and SAS scores (*P* = 0.011).

### Voxel-Wise Functional Connectivity Between the Amygdala and the Whole Brain

**Figure 2:**
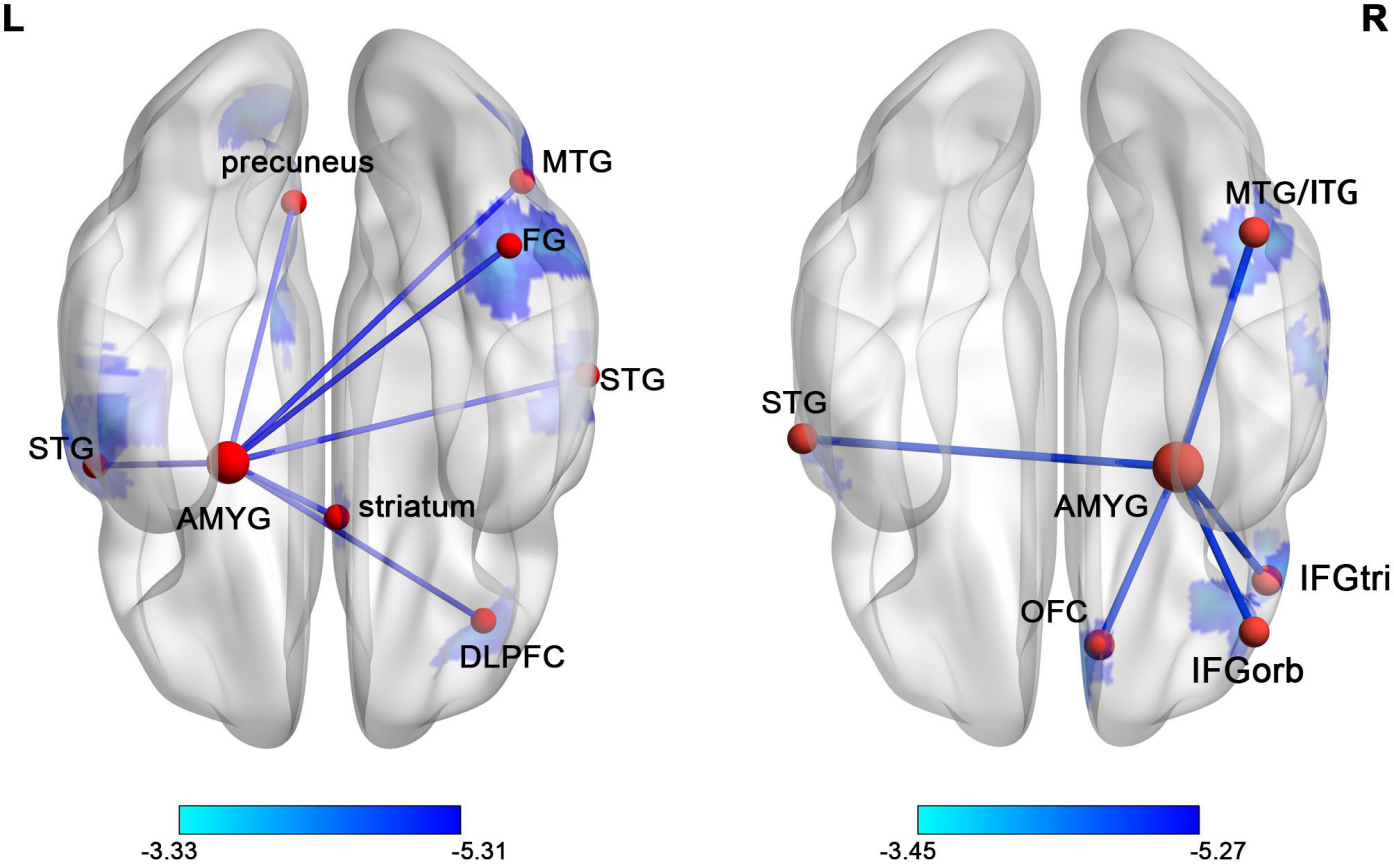
The distribution map of the amygdala FC differences in SNHL was presented in the ventral view. The brain regions with significantly decreased FCs with left or right amygdala were shown in blue. The color bars below show the t scores obtained from the between-group comparison, *P* < 0.05, corrected by FDR. STG, superior temporal gyrus; AMYG, amygdala; MTG, middle temporal gyrus; FG, fusiform gyrus; ITG, inferior temporal gyrus; IFGorb, orbital part of inferior frontal gyrus; IFGtri, triangular part of inferior frontal gyrus; OFC, orbitofrontal cortex.

The voxel-wise comparisons of FC with the left and right amygdala were presented in Figure 2 and Table 2. As a result, SNHL patients exhibited decreased FC with the left amygdala in the bilateral superior temporal gyrus (STG), right middle temporal gyrus (MTG), right dorsolateral pre-frontal gyrus (DLPFC), right fusiform gyrus (FG), right striatum, and left pre-cuneus compared to the healthy controls (*P* < 0.05, corrected by FDR). SNHL patients showed weaker strength of FC with the right amygdala in the left STG, right MTG and inferior temporal gyrus (ITG), right orbitofrontal cortex (OFC), and right triangle and orbital part of the inferior frontal gyrus (IFGtri, IFGorb) compared with the healthy controls (*P* < 0.05, corrected by FDR).

**Table 2:** Differences of FCs with amygdala in SNHL group compared with control group.

**Brain region**	**BA**	**Voxel size**	**Peak MNI coordinates (mm)**			**Peak *t* values**
			**X**	**Y**	**Z**	
**Left amygdala**						
Left superior temporal gyrus	22/41/42	165	−54	0	3	−5.307
Right superior temporal gyrus	41/42	35	60	−21	15	−4.969
Right middle temporal gyrus	37	48	45	−66	9	−4.761
Right dorsolateral frontal cortex	46	31	36	36	27	−4.183
Right fusiform gyrus	37	61	42	−51	−21	−4.456
Right striatum		47	12	15	−6	−4.752
Left precuneus	7	94	−18	−81	45	−4.350
**Right amygdala**						
Left superior temporal gyrus	22/42	46	−60	−6	9	−4.252
Right middle/inferior temporal gyrus	37	108	45	−54	−21	−4.668
Right orbitofrontal cortex	11	40	9	42	−21	−4.443
Right inferior frontal gyrus, orbital part	47	139	45	39	−15	−5.275
Right inferior frontal gyrus, triangular part	45	39	48	27	15	−4.979

*FC, functional connectivity; ROI, region of interest; BA, Brodmann's area; MNI, Montreal Neurological Institute*.

### Tractography of the Bilateral UF

**Figure 3:**
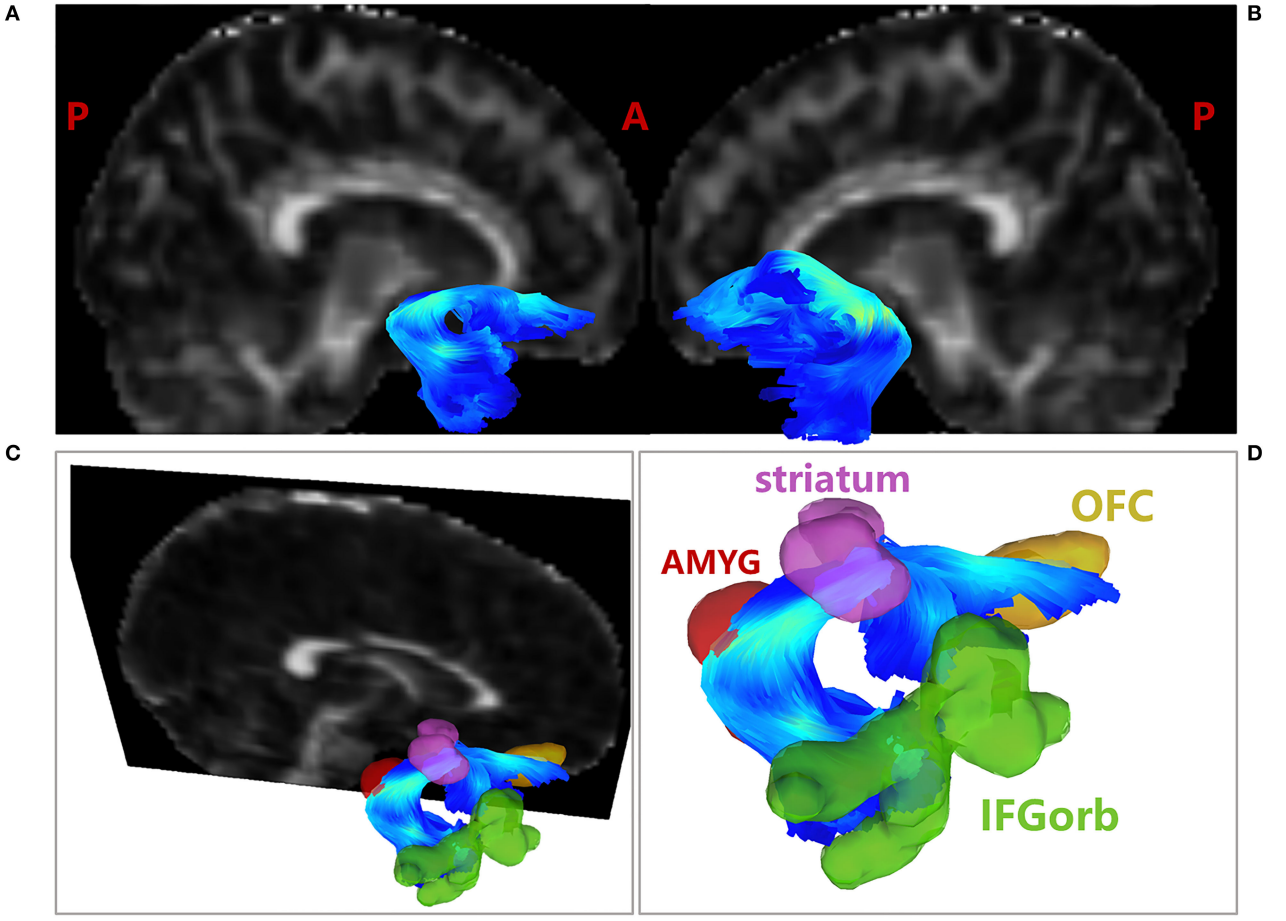
Tractography results of the UF. **(A)** Tractography results of the right UF; **(B)** Tractography results of the left UF; **(C)** Tractography results of the right UF overlaid with decreased FC ROIs, including IFGorb, striatum and OFC; **(D)** Amplified tractography results of the right UF. AMYG in red; OFC in brown; IFGorb in green; striatum in purple. The fibers were color coded for fractional anisotropy (FA) value. AMYG, amygdala; IFGorb, orbital part of inferior frontal gyrus; OFC, orbitofrontal cortex.

After quality control of the diffusion data, one SNHL patient was excluded. As a result, for tractography analyses, there were 37 SNHL patients and 37 healthy controls included in the final tractography analyses. The reconstruction results of the bilateral UF were presented in Figures 3A,B.

Moreover, after overlaying the ROIs found in FC analyses to the fiber space, the right amygdala was found to be structurally connected with the right striatum, IFGorb and OFC by the right UF, as shown in Figure 3D. No direct structural connection between the amygdala and the rest FC decreased ROIs were found through UF. The detailed quantitation results of the bilateral UF were shown in Table 3.

**Table 3:** Diffusion measures of bilateral UF in the SNHL and healthy control groups.

	**Measures**	**SNHL patients**	**Healthy controls**	***P-*value**
		**(*n* = 37)**	**(*n* = 37)**	
Left UF	tract number	3309.32 ± 1111.46	2946.92 ± 1194.18	0.690
	mean length (mm)	65.86 ± 14.61	62.69 ± 14.26	0.805
	mean FA value	0.23 ± 0.03	0.24 ± 0.03	0.058
	mean MD value	0.92 ± 0.07	0.89 ± 0.06	0.015^*^
Right UF	tract number	2957.11 ± 683.56	2825.59 ± 929.12	0.827
	mean length (mm)	92.67 ± 11.17	93.17 ± 11.11	0.256
	mean FA value	0.28 ± 0.03	0.28 ± 0.02	0.656
	mean MD value	0.87 ± 0.06	0.85 ± 0.04	0.952

*Data are represented as mean ± (SD)*.

* *P < 0.05*.

*The comparison was performed after controlling for age, gender, and years of education. UF, uncinate fasciculus; FA, fractional anisotropy; MD, mean diffusion*.

As shown in Table 3, after comparing the track numbers, track length, FA and MD values of the bilateral UF between the SNHL and the healthy control groups, the MD value of the left UF was found to be significantly increased in the SNHL groups (*P* = 0.015), after controlling for age, gender, and years of education.

### Correlational Analyses

**Figure 4:**
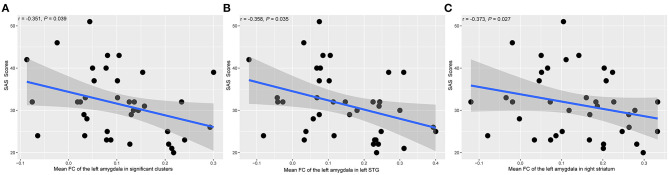
The associations between the amygdala FC alterations and SAS scores in SNHL patients. The presented *P*-values were controlled by the effects of age, gender, and years of education. SAS, Self-Rating Anxiety Scale; STG, superior temporal gyrus. **(A)** The mean FC value between the left amygdala and all the significant clusters. **(B)** The mean FC value between the left amygdala and the left STG. **(C)** The mean FC value between the left amygdala and the right striatum.

Figure 4 showed the scatter plots of all the partial correlations of functional alterations in patients with SNHL. As shown in Figure 4A, SAS scores were negatively associated with the FCs between the left amygdala and all significant voxels from the voxel-wise comparison (*r* = −0.351, *P* = 0.039). SAS scores were also negatively associated with the FCs between the left amygdala and the left STG (*r* = −0.358, *P* = 0.035) and the right striatum (*r* = −0.373, *P* = 0.027), shown in Figures 4B,C. No other significant relations were detected between amygdala FCs and SAS or HAMD scores.

**Figure 5:**
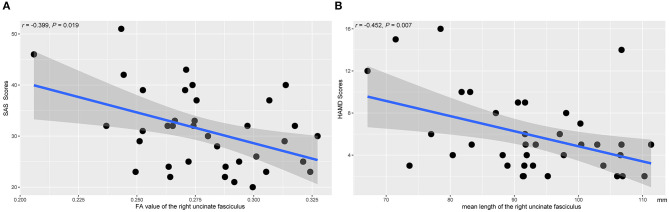
The associations between the diffusion measures of the right UF and the clinical parameters. The presented *P*-values were controlled by the effects of age, gender, and years of education. SAS, Self-Rating Anxiety Scale; HAMD, Hamilton Depression Rating Scale. **(A)** The FA value of the right UF. **(B)** The mean length of the right UF.

Figure 5 showed the scatter plots of all the partial correlations of structural alterations in patients with SNHL. As shown in Figure 5A, SAS scores were negatively associated with the FA values of the right UF (*r* = −0.399, *P* = 0.019). As shown in Figure 5B, HAMD scores were negatively associated with the mean lengths of the right UF (*r* = −0.452, *P* = 0.007). No other significant relations were detected between the diffusion measurements and SAS or HAMD scores.

## Discussion

In this study, wide-spreading suppression of the amygdala FCs were observed in the SNHL patients. By reconstruction of the bilateral UF, the amygdala was found to be structurally connected with several FC decreased regions. Furthermore, significant associations were found between the functional and structural connections of the amygdala with anxiety and depression assessment scores in patients with SNHL. Our results indicate that the altered amygdala connectivity might be related to the negative moods in SNHL.

The amygdala is known to be a crucial structure involved in several emotional functions and related to several emotional impairments, including anxiety and depression. It has been reported that hearing loss causes abnormal neural responses in the amygdala (Husain et al., 2014; Sheppard et al., 2014; Chen et al., 2016). In our study, suppressed FCs between the amygdala and the auditory cortex, striatum, multimodal processing areas, and frontoparietal control areas were found in the SNHL patients. A recent neuroimaging study has revealed the decreased response in the amygdala to the emotional stimuli in hearing loss patients, suggesting that the long-term hearing loss might lessen the acoustic or/and the emotional valence information by impacting the reported connectivity schema between the auditory cortex and amygdala (Husain et al., 2014). A proposed putative causal model underlying the hearing loss-related emotional disorders suggests that the dysfunction in the amygdala, as the neural mediator, leads to negative emotions through the auditory-amygdala dysconnectivity (Rutherford et al., 2018). However, due to the small sample size of the studies, it is difficult to determine whether the hypo-connectivity is a neural alteration or is a reflection of hearing loss in SNHL patients. Future studies are warranted to clarify the underlying neuropathology of these findings.

Additionally, the OFC, amygdala, and ventral striatum are essential components of the central reward system. The OFC is implicated in encoding the stimulus reward values (O'doherty, 2004), and also take part in reward prediction in coordination with the amygdala and ventral striatum (O'doherty et al., 2002). Reduced activation to wins and FC within the reward system have been documented and appeared to be associated with the severity of depression (Satterthwaite et al., 2015). The OFC-striatal circuit was reported abnormally activated in obsession-induced anxiety (Figee et al., 2011). Lower activation elicited by the social reward in the amygdala has also been reported in obsessive-compulsive disorder (Blair et al., 2008), generalized anxiety disorder (Ottaviani et al., 2012), and panic disorder (Cannistraro et al., 2004). The previous perspective supported that the anxiety and depression disorders might be related to the functional reorganization or maladaptive plasticity in the neural circuits related to the reward behavior (Insel and Wang, 2010). Hence, the changed FC among these reward-related regions in the current study might interpret the elevated depressive and anxious states in the patients with SNHL.

The striatum, especially the ventral striatum, is a recipient of the motivational effects of the emotional stimuli from the amygdala, and afterward sends the projections to the regions implicated in the behavioral expression (Phelps et al., 2001). The striatum, together with the amygdala, produces the affective states. In the current study, the SNHL weakened the temporal synchrony between the amygdala and striatum. Such abnormality might eventually contribute to the aberrant response to emotionally significant stimuli or even emotional deficits in SNHL.

Attentional allocation participates in emotional regulation by shifting the attention away from or toward the emotional stimuli dependent on the frontoparietal control areas (Gross, 1998). The emotional regulation might be hindered by the dysfunction of attentional control regions, such as DLPFC, pre-cuneus and ventral pre-frontal cortex (vPFC) involving the IFGorb and IFGtri (Ferri et al., 2016; Rutherford et al., 2018). Reappraisal through the interactions between these cognitive control areas and the amygdala is associated with emotional processing (Ochsner and Gross, 2005; Eippert et al., 2007; Ferri et al., 2016). The decreased amygdala FC in the frontoparietal might underlie the deficits of the emotional modulation in SNHL, which is postulated to be a fundamental basis of the anxiety and emotional disorders (Campbell-Sills and Barlow, 2007).

Furthermore, existing studies have revealed the abnormality of the neural response to the emotional information related to the auditory modality in hearing loss. One of the questions is whether the hearing loss disturbs the emotional processing presented in other perceptual modalities. Our voxel-wise amygdala FC might give a promising understanding of this question. Decreased FC of the amygdala in high-level visual cortex (i.e., fusiform gyrus), and several regions implicated in the visual perception or multimodal integration (i.e., MTG, ITG) (Molholm et al., 2006) among the patients with SNHL possibly resulted from that laborious listening consumed more cognitive resources (Rutherford et al., 2018).

As shown in Figure 3, the shape and trajectory of the UF are highly consistent with the template (Yeh, 2020) and previous literature (Maier-Hein et al., 2017; Park et al., 2019; Sanvito et al., 2020). The UF is the major white matter tract connecting the ventral pre-frontal cortex and the amygdala (Koch et al., 2017), which takes a crucial part of the bidirectional communication within the amygdala-vPFC circuit. However, it is unclear how the structural connectivity of the UF relates to amygdala related emotional impairments. Our results also showed that the FC decreased regions, including the right striatum, IFGorb and OFC, were connected to the right amygdala through the right UF. Besides, the amygdala-striatal hypo-synchronization was negatively associated with the anxiety levels in SNHL, while the FA values of the right UF were also negatively correlated to SAS scores. Our results produced structural evidence of actual anatomical connections between the amygdala and the detected altered FC regions. Moreover, these white matter alternations might contribute to the weaker FCs and drive more significant anxiety and depression levels in SNHL.

As shown in Figure 4, negative FCs were detected in several participants. Negative FC, also called anti-correlation, has been reported since the very beginning of the rs-fMRI technique (Biswal et al., 1995). It represents the negative correlation of the time curses between two brain regions or voxels. Several studies have proved the functional significance of the negative FC (Schwarz and Mcgonigle, 2011; Zhang et al., 2016) and negative FCs were reported between the amygdala and the infralimbic cortex (Liang et al., 2012). In the current study, we retained the individually negative FCs, not to mention that all the reported FC altered areas demonstrated positive FCs at the group level.

The major strength of the current study is that both rs-fMRI and DTI techniques were used to detect structural and functional alterations of the amygdala and their relations to emotional deficits in SNHL patients. Several FC decreased regions were found to be structurally connected through the UF, denoting actual anatomical connections exist between the amygdala and these regions. The increased MD value of the right UF and the negative correlations between the emotional states and diffusion measurements express fundamental abnormalities that might contribute to the altered FCs between the amygdala and the decreased regions.

### Limitations

Several limitations must be acknowledged. Firstly, although the number of participants in the SNHL and control groups was more than 35, it was still not enough to divide the participants into different subgroups according to their emotional states. Secondly, many SNHL patients presented higher depressive and anxious levels simultaneously; the current analyses could not determine the specific neural mechanisms underlying the depressive or anxious emotions in hearing loss. However, the depression is reported highly accompanied by anxiety, suggesting a potential overlap of the neural circuits involving the depression and anxiety (Russo and Nestler, 2013). Finally, due to the cross-sectional design, it is impossible to determine the temporal causality between emotional deficits and hearing loss. A future longitudinal study with a larger sample size might further address this question.

## Conclusions

In conclusion, SNHL caused widespread amygdala FC decreased regions, which were also found to be structurally connected to the amygdala through the uncinate fasciculus. The disturbance of the amygdala related interactions was related to abnormal emotional states in SNHL patients. These findings provided insights, from both anatomical and functional, into the neural loop mechanisms underlying the hearing loss-related emotional impairments.

## Data Availability Statement

The datasets generated for this study are available on request to the corresponding author.

## Ethics Statement

The studies involving human participants were reviewed and approved by the Ethics Committee of Affiliated Zhongda Hospital of Southeast University. The patients/participants provided their written informed consent to participate in this study.

## Author Contributions

G-JT was the study chair and principal investigator. T-YT, YL, and G-JT designed and carried out the study. T-YT and YL drafted the initial manuscript. YL and JZ recruited the participants. T-YT and YL analyzed MRI data. YJ, S-HJ, and G-JT provided extensive critical insights and revisions of all drafts of the manuscript. All authors contributed to the final version of the manuscript.

## Conflict of Interest

The authors declare that the research was conducted in the absence of any commercial or financial relationships that could be construed as a potential conflict of interest.

## Abbreviations

BA: Brodmann's area
DLPFC: dorsolateral pre-frontal gyrus
DTI: diffusion tensor imaging
FC: functional connectivity
FG: fusiform gyrus
HAMD: Hamilton Depression Rating Scale
HL: hearing level
IFGorb: orbital part of inferior frontal gyrus
IFGtri: triangular part of inferior frontal gyrus
FA: fractional anisotropy
MD: mean diffusion
MNI: Montreal Neurological Institute
MRI: magnetic resonance imaging
MTG: middle temporal gyrus
OFC: orbitofrontal cortex
PTA: pure tone audiometry
ROI: region of interest
SAS: Self-Rating Anxiety Scale
SNHL: sensorineural hearing loss
STG: superior temporal gyrus
rs-fMRI: resting state fMRI
UF: uncinate fasciculus.
